# C-Reactive Protein Trajectories by Summary Metric Across the Coronavirus-2019 Period: A 16-Year Interrupted Time-Series Analysis (2008–2023)

**DOI:** 10.3390/diagnostics16071081

**Published:** 2026-04-03

**Authors:** Jeong Su Han, Bo Kyeung Jung, Jae-Sik Jeon, Jae Kyung Kim

**Affiliations:** 1 Department of Biomedical Laboratory Science, College of Health Sciences, Dankook University, Cheonan-si 31116, Republic of Korea; jshan1162@naver.com (J.S.H.); zenty87@naver.com (J.-S.J.); 2 Department of Laboratory Medicine, College of Medicine, Dankook University, Cheonan-si 31116, Republic of Korea; lovegodmother@hanmail.net

**Keywords:** C-reactive protein, COVID-19, harmonic mean, inflammation, long-term trends, stability

## Abstract

**Background/Objectives**: The clinical utility of summarizing long-term C-reactive protein (CRP) trends with a single mean remains unclear. We systematically characterized annual changes in CRP test volume and CRP level distributions using large-scale laboratory data collected at Dankook University Hospital (2008–2023) across the coronavirus 2019 pandemic period. **Methods**: Overall, 1,845,258 CRP values were analyzed; annual arithmetic, harmonic, and geometric means were calculated; long-term trends were assessed using weighted least squares (WLS) regression weighted by annual test volume; and temporal changes around the pandemic period were examined using a WLS-based interrupted time-series (ITS) segmented model with a prespecified 2020 break. **Results**: The annual test volume rose from 2008 to 2013 and 2019, dropped in 2020, increased in 2022, and declined in 2023. The arithmetic mean showed no long-term trend, whereas the harmonic and geometric means declined. ITS models exhibited no statistically significant immediate level-change term in 2020; however, post-2020 slope changes indicated a decline in the arithmetic mean and attenuation of the prior decline in the harmonic mean. As only four annual observations were available after 2020, these post-2020 trend estimates should be interpreted cautiously. **Conclusions**: Within this single-center tertiary-care dataset, different CRP summary measures showed different long-term patterns and post-2020 trend changes, without evidence of an abrupt shift in 2020, suggesting stratum-specific shifts that may be invisible to arithmetic mean-based surveillance. These findings are best interpreted as institution-specific and hypothesis-generating, and broader interpretive or operational implications require validation in multicenter settings with differing case-mix and care structures.

## 1. Introduction

C-reactive protein (CRP) is a representative acute-phase reactant produced in the liver, rising rapidly in response to infectious and non-infectious inflammatory stimuli [1]. In clinical practice, CRP is an important marker for differentiating infectious diseases, evaluating disease severity, and monitoring treatment response; marked elevations are typical in bacterial infections, whereas relatively modest increases are often observed in viral or chronic inflammatory conditions, thereby aiding diagnostic and therapeutic decision-making [2]. However, CRP values tend to cluster at low levels, with a subset of results forming a long tail at higher concentrations, suggesting that reliance on a single mean may be insufficient to capture subtle changes in low-concentration ranges or quantify the burden of high-concentration events across years [3]. Thus, analyzing long-term trends requires summary approaches that simultaneously address multiple segments of the distribution [4].

Accumulated CRP data from hospital laboratories reflect biological responses and operational factors in healthcare utilization, including testing indications, patient demographics, healthcare access, repeat-testing patterns, modifications in clinical services, and epidemic events [5]. While interpreting long-term CRP patterns, it is essential to recognize that changes in both population inflammatory status and the structure of testing and utilization may co-occur. However, many previous longitudinal CRP studies have been constrained by short observation periods or limited to specific patient cohorts (e.g., inpatients or critically ill patients), and research characterizing year-to-year variation over extended periods using large-scale data from routine care is limited [6]. Furthermore, traditional analyses have predominantly relied on arithmetic means [7]. However, in studies of right-skewed biomarkers, including population-based CRP data, geometric means have often been reported alongside arithmetic means or other summary measures to better reflect central tendency under log-normal or multiplicative structure [8]. By contrast, the harmonic mean has been rarely used in CRP research; in the present study, it was included as a complementary summary metric because of its potential sensitivity to changes concentrated in the lower range of the distribution.

Since 2020, the coronavirus 2019 (COVID-19) pandemic has markedly influenced healthcare utilization and laboratory testing practices [9]. During this period, factors such as fluctuating epidemic patterns, altered access to healthcare facilities, deferred visits by patients with mild illness, and evolving workflows and test indications have contributed to potential shifts in observed CRP distributions [10]. Nonetheless, prior studies have shown that the COVID-19 pandemic substantially altered laboratory utilization patterns [11], whereas long-term routine-care studies specifically evaluating CRP trajectories across the pandemic period remain limited.

The present study systematically characterized annual changes in CRP test volume and CRP level distributions using large-scale laboratory data collected from 2008 to 2023 at Dankook University Hospital. By examining pre- and post-2020 trajectories and comparing multiple summary metrics within the same dataset, this study provides a robust single-center analysis of how long-term laboratory patterns can vary according to analytic approach. Although the findings should be interpreted in the context of this regional tertiary-care setting, they offer a practical and reproducible framework for future multicenter studies evaluating the broader applicability of laboratory-based CRP surveillance.

## 2. Materials and Methods

### 2.1. Study Design and Population

This single-center retrospective observational study was conducted at Dankook University Hospital—a tertiary-care referral hospital in Cheonan, a major city in Chungcheongnam-do, Republic of Korea, serving both local residents and referred patients from the surrounding region—located in Cheonan, a major city in central Korea near the Seoul metropolitan area. The population of Cheonan was 664,746, and Dankook University Hospital is a large regional hospital with 921 beds. In this study, results from CRP tests performed from 2008 to 2023 were analyzed. All de-identified CRP test results recorded in the electronic medical records during the study period were included, and the analysis was conducted using annually aggregated summary measures of CRP—the arithmetic mean, harmonic mean, and geometric mean—with calendar year as the primary unit of analysis.

### 2.2. Data Collection and Variables

**Figure 1:**
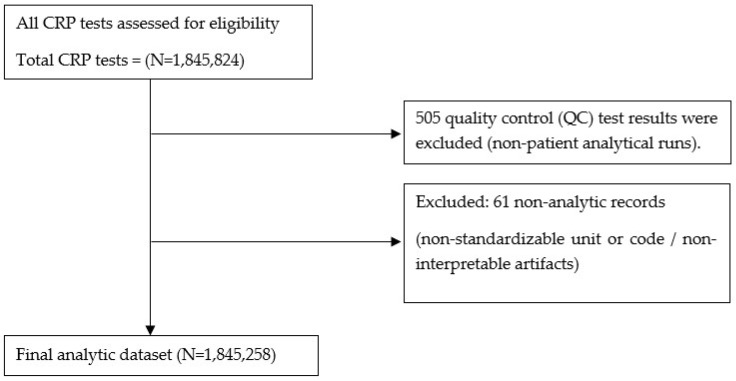
Flow diagram of CRP test records (test-level) included for annual analyses (2008–2023). All CRP tests were initially assessed for eligibility (*N* = 1,845,824). Quality control (QC) results were excluded as non-patient analytical runs (*n* = 505). Records with non-interpretable artifact values and those with ambiguous units or codes that precluded standardization were additionally excluded (*n* = 61). The final analytic dataset included 1,845,258 test records. Abbreviations: CRP, C-reactive protein.

A total of 1,845,824 CRP test records were identified during the study period (Figure 1). Quality control (QC) results—tests run on artificial control materials rather than patient-derived specimens for routine instrument and reagent performance checks—were excluded because they do not represent patient CRP levels (*n* = 505). Records with non-interpretable artifact values and those with ambiguous units or codes that precluded standardization were additionally excluded (*n* = 61). The remaining 1,845,258 records were included in the final analysis. For each test record, the calendar year of specimen collection and the CRP result (mg/dL) were extracted and analyzed. In addition, the dataset was screened for implausible analytical values outside the measurement range of the assay. No CRP values inconsistent with the analytical limits of the assay were identified.

### 2.3. CRP Measurement

All CRP assays were performed on the Cobas 8000 modular analyzer series (Roche Diagnostics, Mannheim, Germany) using dedicated reagents (Tina-quant C-Reactive Protein Gen.3, Roche Diagnostics, Mannheim, Germany) according to an automated immunoturbidimetric method. The analytical range was 0.03–35 mg/dL, and the coefficient of variation (CV) was <3%. When results exceeded the upper limit of the primary measuring range, the analyzer’s automatic 1:2 dilution and repeat measurement procedure was applied, allowing reporting within an extended range (0.06–70 mg/dL). In this study, the final reported values recorded in the laboratory information system were used for analysis. All CRP values were analyzed exactly as numerically recorded in the laboratory information system (LIS), with no additional imputation (substitution with LoQ/2) applied. After clotting, blood samples were centrifuged immediately to separate serum, stored at 2–8 °C, and analyzed within 24 h. Internal and external quality-control procedures were maintained throughout the study period to ensure analytical consistency across the years.

### 2.4. Statistical Analysis

Annual arithmetic, harmonic, and geometric mean CRP concentrations were calculated by calendar year. The geometric mean was computed at the test level as exp(mean(ln(CRP))) using the natural logarithm (all CRP values were positive). As the minimum reportable CRP value of the assay was 0.03 mg/dL, no zero values were present in the dataset; therefore, both geometric and harmonic means could be computed directly without additional transformation. The harmonic mean was computed as *n*/Σ(1/CRP) and was used alongside the arithmetic mean to compare center-versus-tail changes in the distribution. Long-term trends were evaluated using weighted least squares (WLS) regression, with annual test volume (*N*) used as the analytic weight. As a part of sensitivity analysis, quadratic models including centered year and its squared term were additionally fitted for the annual arithmetic, harmonic, and geometric mean CRP values to assess possible non-linearity. We prespecified 2020 as the primary interruption point because it corresponded to the onset of the COVID-19 pandemic and the beginning of major changes in healthcare utilization, testing indications, infection-control practices, and respiratory infection epidemiology. Accordingly, an interrupted time-series (ITS) segmented regression model was fitted for 2008–2019 vs. 2020–2023 periods using weighted least squares (WLS), with the annual number of tests (Tests) applied as analytic weights. The model included a continuous time term (β_1_; pre-2020 slope), a post-2020 indicator term (β_2_; immediate level change at 2020), and a time-after-2020 term (β_3_; change in slope after 2020).

To assess the robustness of the findings to breakpoint specification, we additionally performed sensitivity analyses using alternative interruption points at 2019 and 2021 while retaining the same weighted least squares ITS framework and analytic weights. As the post-interruption annual segment was short, these analyses were intended to evaluate the stability of the estimated level- and slope-change parameters under nearby alternative breakpoint definitions rather than to identify a single definitive interruption year. To evaluate the potential influence of extreme repeat testing, we performed an additional sensitivity analysis excluding individuals with more than 300 CRP measurements over the entire study period. After this exclusion, annual arithmetic, harmonic, and geometric mean CRP concentrations were recalculated, and both the weighted least squares long-term trend models and the 2020-interruption ITS models were refitted using the same analytic framework as in the primary analysis. This analysis was designed to assess whether the principal temporal patterns were disproportionately driven by a very small subset of heavily monitored patients. We also conducted a sensitivity analysis excluding 2008, the initial low-volume year, by refitting the long-term trend and 2020-interruption ITS models under the same weighted least squares framework.

To account for multiple comparisons across the three parallel ITS summary metrics, *p*-values for corresponding model parameters were additionally adjusted across the arithmetic, harmonic, and geometric mean models using the Bonferroni method. Benjamini–Hochberg (BH) adjusted *p*-values were also examined as a sensitivity check. Regarding post-2020 trajectory differences, multiplicity-adjusted inference was specifically emphasized for the level-change (β_2_) and slope-change (β_3_) terms.

To obtain valid inference under potential heteroskedasticity and serial correlation in annual time-series data, robust standard errors were computed using the Newey–West heteroskedasticity- and autocorrelation-consistent (HAC) estimator (lag = 1). As the time series consisted of annual observations with a limited number of time points, a lag of 1 was selected to account for potential first-order autocorrelation while avoiding over-parameterization of the variance estimator in a short time series. Counterfactual projections for 2020–2023 assumed continuation of the pre-2020 trend. All analyses were conducted in R software (version 4.5.1; The R Foundation for Statistical Computing, Vienna, Austria), and statistical significance was defined as a two-sided *p*-value < 0.05. The R scripts used for the statistical analyses are provided in File S1.

## 3. Results

### 3.1. Annual CRP Test Volumes and Mean Concentrations (2008–2023)

A total of 1,845,824 CRP test records were identified. The exclusion workflow for quality-control results (*n* = 505) and non-analytic/non-standardized entries (*n* = 61) is summarized in Figure 1, which also provides an overview of the test-count-based CRP dataset used for the annual analyses (2008–2023). Annual CRP test volume increased sharply from 5015 tests in 2008 to 108,431 tests in 2013, exceeding 100,000 tests per year. This upward trend continued, reaching 153,581 tests in 2019. Test volume declined to 143,811 in 2020, increased again to 169,697 in 2022, and then decreased to 112,499 in 2023 (Table 1).

**Figure 2:**
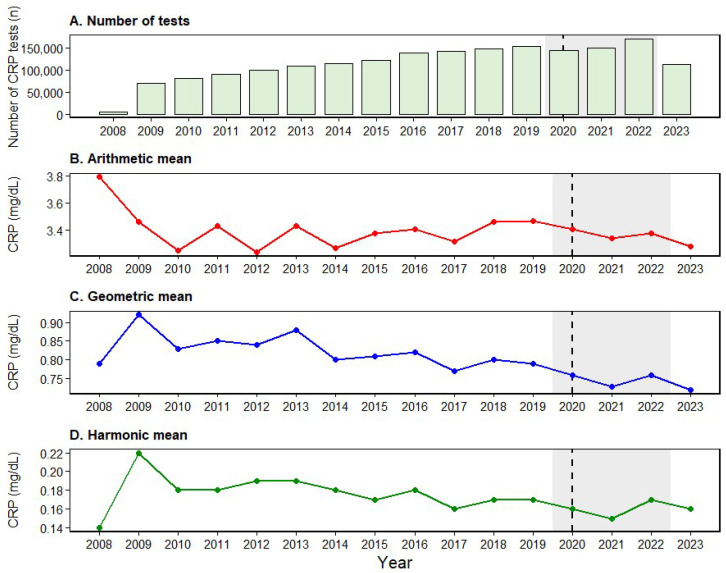
Annual trends in CRP summary statistics and test volume, 2008–2023. (**A**) Annual number of CRP tests. (**B**–**D**) Annual arithmetic mean, geometric mean, and harmonic mean CRP concentrations (mg/dL), respectively. The dashed vertical line at 2020 indicates the onset of the COVID-19 pandemic, and the gray shaded region denotes the pandemic period (2020–2022). Abbreviations: CRP, C-reactive protein.

Overall, CRP concentrations ranged from the minimum reportable value of 0.03 mg/dL to a maximum observed value of 69.06 mg/dL. The median was 0.79 mg/dL, and the interquartile range (Q1–Q3) was 0.20–4.18 mg/dL (IQR, 3.98 mg/dL). The annual arithmetic mean CRP concentration ranged from 3.24 to 3.79 mg/dL. Specifically, it decreased from 3.79 mg/dL in 2008 to 3.24 mg/dL in 2012, remained at approximately 3.46–3.47 mg/dL in 2018–2019, and reached 3.28 mg/dL in 2023. The annual harmonic mean ranged from 0.14 to 0.22 mg/dL, with the highest value in 2009 (0.22 mg/dL) and the lowest in 2008 (0.14 mg/dL). The annual geometric mean ranged from 0.72 to 0.92 mg/dL, peaking in 2009 (0.92 mg/dL) and reaching its lowest level in 2023 (0.72 mg/dL) (Table 1; Figure 2).

In the overall cohort, 156,790 individuals underwent CRP testing once, and 49,462 underwent testing twice. A small subset of records reflected frequent repeat measurements within the same individual, with a maximum of 599 CRP tests per patient (Table S1).

### 3.2. Long-Term Temporal Trends in CRP Concentrations

**Figure 3:**
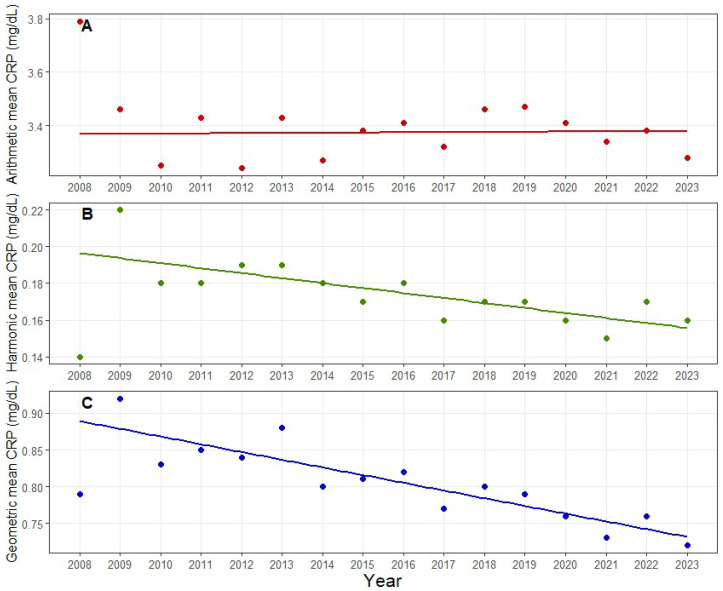
Divergent long-term temporal trends in annual CRP summary measures (2008–2023). (**A**) Annual arithmetic mean CRP, (**B**) annual harmonic mean CRP, and (**C**) annual geometric mean CRP are presented as data points; the arithmetic mean remained stable over time, whereas both the harmonic and geometric means showed significant downward trends, and CRP concentrations are expressed in mg/dL. Abbreviations: CRP, C-reactive protein.

Over the 2008–2023 period, long-term annual trends in CRP summary metrics differed by the statistic used (Table 2). The arithmetic mean CRP showed no significant temporal change (β = +0.000765 mg/dL/year, *p* = 0.880). In contrast, both the harmonic mean and geometric mean CRP declined significantly over time (harmonic mean: β = −0.002716 mg/dL/year, *p* < 0.001; geometric mean: β = −0.010504 mg/dL/year, *p* < 0.001) (Figure 3).

These long-term patterns were materially unchanged in a sensitivity analysis excluding individuals with more than 300 CRP measurements across the study period: the arithmetic mean remained stable over time (β = +0.000648 mg/dL per year, *p* = 0.899), whereas the harmonic mean (β = −0.002506, *p* < 0.001) and geometric mean (β = −0.010307, *p* < 0.001) continued to show significant negative trends (Table S2).

These long-term patterns were also preserved in a sensitivity analysis excluding 2008, the initial low-volume year (Table S3). After removal of 2008, the arithmetic mean remained stable over time (β = +0.001385 mg/dL per year, *p* = 0.786), whereas the harmonic mean (β = −0.002799, *p* < 0.001) and geometric mean (β = −0.010649, *p* < 0.001) continued to show significant negative trends.

Quadratic sensitivity analyses did not support reasonable non-linearity for any annual CRP summary metric, and the main interpretation remained unchanged (Table S4).

### 3.3. Post-2020 Changes in Annual CRP Trajectories: Interrupted Time-Series Analysis (2008–2023)

**Figure 4:**
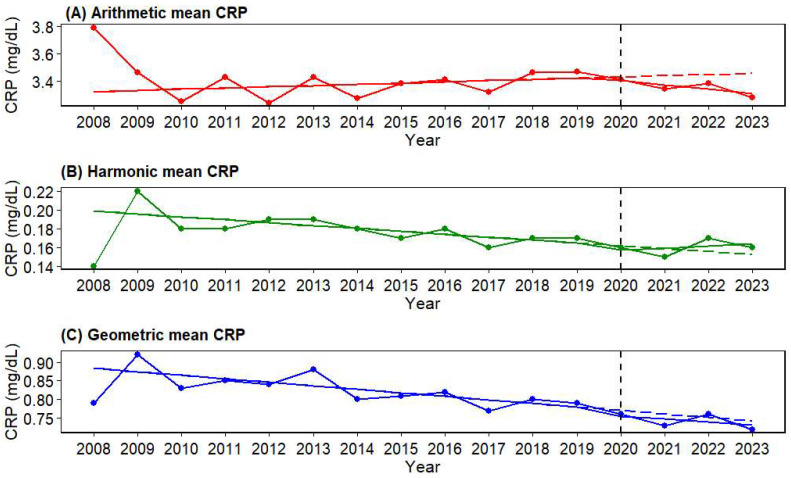
Interrupted time-series (ITS) trajectories of annual CRP summary measures with a prespecified 2020 interruption (2008–2023). (**A**) Arithmetic mean CRP (red), (**B**) harmonic mean CRP (green), and (**C**) geometric mean CRP (blue) concentrations (mg/dL) from 2008 to 2023 are presented. Colored points indicate the observed annual values. In each panel, the colored solid line represents the fitted interrupted time-series (ITS) trajectory from a two-segment segmented regression model including time (pre-2020 slope), a post-2020 indicator (immediate level change in 2020), and time after 2020 (post-2020 slope change), whereas the colored dashed line denotes the counterfactual trajectory, defined as the expected post-2020 course had the pre-2020 trend continued without interruption. The vertical dashed line marks the prespecified interruption in 2020. Abbreviations: CRP, C-reactive protein.

Across all three CRP summary metrics (arithmetic, harmonic, and geometric means), the immediate post-2020 level change was not statistically significant (arithmetic mean: β_2_ = −0.0251, 95% CI −0.110 to +0.0596, *p* = 0.572; harmonic mean: β_2_ = −0.00488, 95% CI −0.0154 to +0.00562, *p* = 0.380; geometric mean: β_2_ = −0.0151, 95% CI −0.0387 to +0.00847, *p* = 0.233) (Figure 4); (Table 3). In contrast, post-2020 slope changes differed by metric. To account for multiple comparisons across the three parallel ITS summary metrics, multiplicity-adjusted *p*-values were additionally examined for corresponding model parameters. For the post-2020 slope-change term (β_3_), the arithmetic mean result remained significant after Bonferroni correction (adjusted *p* = 0.0165), whereas the harmonic mean result, although nominally significant in the unadjusted analysis (raw *p* = 0.0409), no longer met the corrected significance threshold (Bonferroni-adjusted *p* = 0.1227; BH-adjusted *p* = 0.0614). The geometric mean result remained non-significant (Table S5).

For the arithmetic mean CRP, there was no significant pre-pandemic trend (β_1_ = +0.00901 mg/dL/year, *p* = 0.219), whereas the slope shifted significantly downward after 2020 (β_3_ = −0.0414 mg/dL/year, 95% CI −0.0654 to −0.0174, *p* = 0.00549). Accordingly, the post-pandemic net slope was estimated as −0.0324 mg/dL/year (95% CI −0.0515 to −0.0133; *p* = 0.000893). Relative to the counterfactual trajectory, the deviation in 2023 was −0.149 mg/dL, and the cumulative deviation over 2020–2023 was −0.349 mg/dL·year, indicating that arithmetic mean CRP remained lower than expected after the pandemic.

For the harmonic mean CRP, a significant declining trend was observed pre-pandemic (β_1_ = −0.00311 mg/dL/year, *p* = 0.00780), but the post-2020 slope change term was significantly positive (β_3_ = +0.00557 mg/dL/year, 95% CI +0.000804 to +0.0103, *p* = 0.0409), suggesting attenuation of the prior decline. The post-pandemic net slope (β_1_ + β_3_) was +0.00246 mg/dL/year, but this was not statistically significant (*p* = 0.250). The deviation from the counterfactual was +0.0118 mg/dL in 2023, and the cumulative deviation over the 2020–2023 period was +0.0139 mg/dL·year, indicating that harmonic mean CRP was modestly higher than expected (i.e., declined less) after the pandemic.

For the geometric mean CRP, a clear pre-pandemic decline was observed (β_1_ = −0.00943 mg/dL/year, *p* < 0.001), and there was no significant additional slope change after 2020 (β_3_ = +0.00167 mg/dL/year, 95% CI −0.00876 to +0.0121, *p* = 0.760). The post-pandemic net slope was −0.00776 mg/dL/year, which was not statistically significant (*p* = 0.109). Relative to the counterfactual, the deviation in 2023 was −0.0101 mg/dL, and the cumulative deviation during 2020–2023 was −0.0506 mg/dL·year, smaller in magnitude than that observed for the arithmetic mean.

Results of sensitivity analyses using alternative interruption points at 2019 and 2021 are shown in Table S6. Across breakpoint specifications, the immediate level-change term (β_2_) remained non-significant for all three CRP summary metrics. For the arithmetic mean, the slope-change term (β_3_) remained negative across all three breakpoint specifications (2019: −0.0444, 95% CI −0.0645 to −0.0243; 2020: −0.0414, 95% CI −0.0654 to −0.0174; 2021: −0.0340, 95% CI −0.0800 to +0.0120), although statistical significance was not retained for the 2021 specification. For the harmonic mean, β_3_ remained positive across all breakpoint specifications (2019: +0.00264, 95% CI −0.00165 to +0.00694; 2020: +0.00557, 95% CI +0.000804 to +0.0103; 2021: +0.00896, 95% CI −0.00100 to +0.0189), and was statistically significant only in the primary 2020 model. For the geometric mean, no statistically significant slope-change term was observed under any breakpoint specification.

In a sensitivity ITS analysis excluding individuals with more than 300 CRP measurements over the study period, the immediate level-change term at 2020 (β_2_) remained non-significant across all three summary metrics (Table S7). The arithmetic mean showed a negative post-2020 slope-change estimate (β_3_ = −0.0399, *p* = 0.268) and post-2020 net slope (β_1_ + β_3_ = −0.0307, *p* = 0.377), while the harmonic and geometric means retained significant pre-2020 declines (β_1_ = −0.00262, *p* = 0.016; and β_1_ = −0.00890, *p* = 0.005, respectively) with non-significant post-2020 slope-change terms. The 2023 deviations were −0.151, +0.00955, and −0.0136 mg/dL for the arithmetic, harmonic, and geometric means, respectively (Table S8).

In an additional sensitivity ITS analysis excluding 2008, the initial low-volume year, the overall pattern remained similar (Tables S9 and S10). The arithmetic mean retained a significant negative post-2020 slope change (β_3_ = −0.042721, *p* = 0.005) and a negative post-2020 net slope (β_1_ + β_3_ = −0.032372, *p* = 0.001). The harmonic mean continued to show a significant pre-2020 decline and a positive post-2020 slope-change term, although the post-2020 net slope was not significant. For the geometric mean, the pre-2020 decline remained significant, whereas the post-2020 slope change was non-significant.

**Table 3:** Two-segment interrupted time-series (ITS) segmented regression of annual CRP summary measures with a prespecified 2020 interruption (2008–2019 vs. 2020–2023).

Metric	Parameter	Estimate (β)	95% CI	*p*-Value
Arithmetic mean	β_1_: Pre-2020 slope	+0.00901	−0.00461 to +0.0226	0.219
	β_2_: Level change at 2020	−0.0251	−0.110 to +0.0596	0.572
	β_3_: Slope change after 2020	−0.0414	−0.0654 to −0.0174	0.00549
	Post-2020 net slope (β_1_ + β_3_)	−0.0324	−0.0515 to −0.0133	0.000893
Harmonic mean	β_1_: Pre-2020 slope	−0.00311	−0.00502 to −0.00120	0.00780
	β_2_: Level change at 2020	−0.00488	−0.0154 to +0.00562	0.380
	β_3_: Slope change after 2020	+0.00557	+0.000804 to +0.0103	0.0409
	Post-2020 net slope (β_1_ + β_3_)	+0.00246	−0.00173 to +0.00666	0.250
Geometric mean	β_1_: Pre-2020 slope	−0.00943	−0.0133 to −0.00559	<0.001
	β_2_: Level change at 2020	−0.0151	−0.0387 to +0.00847	0.233
	β_3_: Slope change after 2020	+0.00167	−0.00876 to +0.0121	0.760
	Post-2020 net slope (β_1_ + β_3_)	−0.00776	−0.0173 to +0.00173	0.109

Annual arithmetic, harmonic, and geometric mean CRP concentrations (mg/dL) were modeled using a two-segment ITS framework with 2020 as the prespecified interruption. The model included a continuous time term (β_1_; pre-2020 slope), a post-2020 indicator (β_2_; immediate level change at 2020), and a time-after-2020 term (β_3_; change in slope after 2020). The post-2020 net slope was calculated as β_1_ + β_3_ and is reported with its 95% confidence interval. Weighted least squares (WLS) regression was fitted using annual test volume (*N*) as analytic weights.

## 4. Discussion

This study, based on CRP laboratory results from a single tertiary-care hospital between 2008 and 2023, revealed that year-to-year changes are difficult to summarize as a simple increase or decrease in a single mean. Given that the CRP distribution has an asymmetric structure wherein low concentrations are densely clustered and high concentrations form a long right tail, the arithmetic mean is strongly influenced by the upper tail [12,13,14]. By contrast, geometric and harmonic means are more sensitive than the arithmetic mean to changes in the center and low-concentration range of the distribution [15]. Accordingly, even within the same dataset, the magnitude and direction of observed change can differ depending on the summary metric used, and a single indicator may be insufficient to capture distribution-stratum-specific changes. The central finding of this study is not that values shifted uniformly in a single direction, rather that long-term signals can be identified by examining how the upper tail and the central or low-concentration regions evolve at differing rates and in distinct directions. Specifically, a single mean value does not adequately account for the persistence of tail burden alongside concurrent shifts in the distribution’s center. In contrast, employing multiple summary metrics concurrently provides a clearer, more interpretable view of changes in the distributional structure.

In prior studies, CRP has primarily been treated as a representative marker linked to chronic inflammatory burden, cardiometabolic risk, and infection severity [16,17,18]. Studies using population-based data have consistently demonstrated that population-level determinants such as body mass index, lifestyle, metabolic status, and socioeconomic factors are strongly coupled with CRP distribution [19,20]. In laboratory-based long-term data, patient composition, testing indications, healthcare utilization, and the repeat-testing structure may vary over time. For example, the decline in test volume in 2023 suggests that annual means may reflect not only changes in disease burden but also the combined influence of healthcare access, referral patterns, and shifts in patient composition. As annual aggregation was performed using consistent electronic extraction criteria, the observed decline likely reflects the actual clinical operating environment. Factors such as changes in clinical pathways for CRP ordering, modifications in the outpatient–inpatient population mix, and revisions to testing indications may have contributed to the reduced test volume. This study’s contribution centers on demonstrating that long-term laboratory data trends do not reflect changes in individual inflammatory physiology alone; rather, they are composite signals shaped by concurrent shifts in operational clinical context—including prescribing indications, patient demographics, and repeat-testing patterns. As annual CRP test volume may have reflected both changes in underlying inflammatory burden and shifts in institutional expansion, referral patterns, service mix, and ordering practices, the yearly summaries are best interpreted as institution-level composite signals, with broader surveillance or operational implications requiring confirmation in future multicenter studies across diverse hospital settings. Therefore, interpreting mean changes strictly as “inflammation increased or decreased” risks conflating divergent trends across different segments of the distribution into an oversimplified conclusion. Valid interpretation requires identifying which distribution stratum has shifted, thereby distinguishing whether the underlying changes are biological, operational, or a combination of both. Given CRP’s right-skewed distribution with a pronounced upper tail, prior research has adopted methods presenting geometric means and medians alongside arithmetic means to more accurately reflect central tendencies [21,22,23]. Within this framework, the present study highlights that the selection of summary metrics can reasonably influence the interpretation of long-term distributional signals—a methodological consideration that is frequently overlooked in laboratory surveillance reports but remains critical for safe and accurate interpretation.

Although directly comparable long-term hospital-based studies employing parallel CRP summary metrics remain limited, evidence from clinical and population-based settings in the United States and the United Kingdom suggests that CRP distributions are shaped not solely by inflammatory biology, but also by case mix, patterns of healthcare utilization, and population characteristics such as race/ethnicity and socioeconomic status [24,25]. Within this broader context, our findings align with the literature indicating that CRP trends should be interpreted with attention to both distributional structure and clinical context, rather than as reflecting a simple uniform change in inflammatory burden.

This interpretation was further supported by sensitivity analyses excluding individuals with more than 300 CRP measurements over the study period. In the analysis, the long-term annual patterns were essentially unchanged: the arithmetic mean remained stable, whereas the harmonic and geometric means continued to decline significantly. This consistency indicates that the divergent long-term behavior across CRP summary metrics was not driven solely by a small subset of extremely frequent testers, but instead reflected a broader redistribution of the annual CRP distribution. Similar findings were obtained after excluding 2008, the initial low-volume year, supporting the robustness of the long-term divergence across summary metrics.

During the pandemic, CRP was widely used to assess disease severity and monitor patients. This period saw significant changes in laboratory indicator observation environments as healthcare access, outpatient–inpatient structures, consumables, infectious disease patterns, and clinical protocols evolved simultaneously [26,27]. Research on testing operations and efficiency during the pandemic has demonstrated that operational shifts can affect test composition and performance [28]. Rather than asserting that CRP changed abruptly due to the pandemic, this study suggests that the altered clinical environment led to distinct responses across different distributional strata. While the upper tail of CRP values may have remained stable, the central and lower concentrations may have shifted in other directions, highlighting that single-mean-centered analyses may overlook such nuances. These differences also reflect the distinct questions addressed by each model. A single linear trend across 2008–2023 showed no significant long-term change in the arithmetic mean, whereas the annual ITS analysis with 2020 as a prespecified breakpoint suggested a post-2020 change in trajectory. This may indicate reconfiguration following a major external disruption rather than genuine stability. However, because the post-break segment included only four annual observations, the estimated level- and slope-change parameters should be regarded as underpowered and potentially unstable; thus, the post-2020 findings are best interpreted as exploratory. Importantly, after multiplicity adjustment across the three parallel ITS summary metrics, the arithmetic mean post-2020 slope-change signal remained statistically significant, whereas the corresponding harmonic mean signal did not retain significance. This pattern suggests that the arithmetic mean finding was comparatively more robust, while the harmonic mean change should be interpreted more cautiously as a suggestive rather than confirmatory signal. Accordingly, the non-significant β_2_ estimates may reflect either a true absence of an immediate level shift or limited power to detect one. This caution was further supported by the repeat-testing sensitivity analysis excluding individuals with more than 300 CRP measurements. In the analysis, the post-2020 slope-change estimates for the arithmetic and harmonic means remained directionally similar to those in the primary model, but statistical significance was no longer retained. Sensitivity analyses using alternative breakpoints showed that the post-break decline in the arithmetic mean was broadly preserved with a 2019 breakpoint but weakened with a 2021 breakpoint, indicating that the signal was not entirely dependent on choosing 2020 alone, while also showing that its timing and magnitude were sensitive to breakpoint placement. The corresponding positive slope change in the harmonic mean was most evident only with the 2020 breakpoint, suggesting limited robustness, whereas the geometric mean showed no clear structural break in either the main or sensitivity analyses. Excluding 2008, the initial low-volume year also yielded a similar ITS pattern, suggesting that the principal post-2020 findings were not driven by the earliest year alone. Overall, these findings reinforce that long-term interpretation depends materially on the summary metric selected. Importantly, the counterfactual deviations presented in Table 4 were modest in absolute terms when viewed against commonly used CRP decision thresholds. Thus, their clinical relevance is likely limited in routine individual-patient decision contexts, while still being potentially reasonable as indicators of redistribution in aggregate inflammatory burden at the hospital level.

The repeated-measures structure is fundamental to interpreting distributions of laboratory data. Recent large-scale primary-care-based studies emphasize substantial within-person variability in CRP measurements, cautioning that single measurements may not accurately reflect an individual’s status [29]. In laboratory datasets, repeated tests from the same patient directly influence summary statistics; therefore, annual means capture both patient-level inflammatory burden and variation in testing frequency and clinical context. High-CRP events in the upper tail of the distribution may indicate severe infections or inflammation, whereas middle and low levels may indicate chronic inflammatory burden, mild illness prevalence, or shifts in testing indications [30,31,32]. Notably, repeat-testing frequency per patient varied considerably, reaching a maximum of 599 tests in a single individual over the study period. Such extreme repeat testing is unlikely to represent routine surveillance alone and more plausibly reflects persistent illness, prolonged follow-up, recurrent hospitalization, or closely monitored clinical episodes. Consequently, a small number of highly retested patients could disproportionately affect annual summary values, particularly metrics that are sensitive to upper-tail observations and repeated high-CRP episodes. However, the sensitivity analysis excluding individuals with more than 300 CRP measurements yielded materially similar long-term patterns, indicating that the divergent behavior across summary metrics was not driven solely by these extreme repeat testers. Therefore, in laboratory big data, annual CRP summaries should be interpreted as composites of inflammatory burden and testing intensity, and the choice of aggregation level remains a key determinant of long-term trend interpretation. Presenting multiple summary metrics in parallel supports the interpretation that different strata yield distinct signals, arising from the observational context.

Rather than establishing a general surveillance strategy, this single-center analysis illustrates how the choice of summary metric can materially change interpretation of annual CRP trends within one tertiary-care clinical environment. It advocates shifting the analysis from a single annual mean to distributional strata and interprets the pandemic’s impact by characterizing the nature of change rather than simply noting pre–post mean differences. Placing the selection of summary metrics at the forefront of result interpretation clarifies that defining a representative value effectively sets the parameters for understanding trends in biomarker surveillance. This methodology is broadly applicable to indicators such as CRP, which exhibit asymmetric distributions and clinically reasonable outliers.

Nonetheless, certain limitations must be considered. A critical limitation of the annual interrupted time-series analysis is that the post-2020 segment contained only four annual observations (2020–2023). With such a short post-breakpoint segment, the estimated level-change (β_2_) and slope-change (β_3_) parameters are underpowered and potentially unstable, and may be disproportionately influenced by individual annual observations, particularly 2023. Therefore, the post-2020 slope-related findings should be interpreted as exploratory rather than definitive. In addition, because inference was conducted across three parallel ITS summary metrics, multiplicity-adjusted *p*-values were additionally examined. Under this correction, the arithmetic mean post-2020 slope-change signal remained significant, whereas the corresponding harmonic mean signal did not retain significance, reinforcing a cautious interpretation of the latter. Data were derived from a single institution, and variations in patient demographics, healthcare delivery systems, and testing practices across settings may limit the generalizability of these distributional patterns [33]. In addition, the dataset did not include sufficiently granular year-specific information on the clinical structure of the tested population, such as the proportions of outpatients, hospitalized patients, and ICU patients, the indications for CRP ordering, or potential changes in diagnostic pathways and ordering protocols over time. As these factors may independently influence annual CRP distributions, we could not determine the extent to which the observed shifts reflected changes in inflammatory burden versus changes in case mix, disease severity, or institutional testing practice. Accordingly, the yearly summaries should be interpreted as institution-level composite signals rather than as clinically specific measures of disease activity alone. Future research should aim to confirm reproducibility using multicenter data, incorporate demographic stratification, and integrate variables such as outpatient/inpatient/ICU status, clinical departments, infectious versus non-infectious diagnostic groups, comorbidities, and medication exposure to specify the drivers of stratum-specific shifts. Supplementing current methods with indicators reflecting tail proportion and patient-level summaries would enhance simultaneous monitoring of both distribution centers and extremes. From a stewardship perspective, elucidating clinical contexts in which CRP is ordered and expanding analyses to multicenter and multinational surveillance would strengthen the clinical persuasiveness of interpretation.

In summary, long-term changes in CRP values reflect redistribution across various distributional strata, and the choice of summary metric is pivotal in determining both the meaning of observed change and surveillance strategies. By demonstrating how different representative values yield distinct interpretations within the same dataset, this study provides methodological guidance for adopting multi-dimensional indicator systems that accurately capture both central tendencies and outliers in future CRP surveillance and related research.

## 5. Operational and Clinical Implications

From an operational laboratory perspective, the present findings suggest that annual CRP surveillance should not rely on a single summary metric alone. In this single-center dataset, arithmetic, geometric, and harmonic means captured different aspects of the annual CRP distribution and yielded divergent long-term trajectories, while annual test volume also changed substantially across years. Accordingly, concurrent review of annual test volume with multiple CRP summary metrics may provide a more informative institution-level framework for interpreting whether observed year-to-year changes are more consistent with redistribution across distributional strata than with a uniform shift in CRP values. In practical terms, this approach may assist LIS-based annual monitoring, interpretation of utilization-related change, and contextual assessment of shifts potentially associated with patient mix or CRP ordering practices. These implications should be interpreted as institution-specific and operational, not as direct evidence for patient-level diagnostic thresholds or broad inter-hospital generalizability.

## 6. Conclusions

Based on 1,845,258 CRP test results obtained at a single tertiary-care institution between 2008 and 2023, this study showed that the long-term interpretation of annual CRP patterns can vary materially according to the summary metric selected. Arithmetic, harmonic, and geometric means reflected different strata of the CRP distribution even within the same dataset and accordingly yielded different long-term trajectories. In addition, the annual ITS analysis suggested that post-2020 changes were more consistent with gradual redistribution across the overall distribution than with a clearly detectable abrupt shift. These findings indicate that annual CRP trends may require interpretation beyond a simple reliance on a single summary metric, with consideration of multiple aspects of the distribution. However, these results are most appropriately interpreted as institution-specific and hypothesis-generating. Accordingly, rather than establishing a generalized surveillance strategy, this study provides a practical single-center framework for a more refined interpretation of annual CRP changes in institutional laboratory data. Broader interpretive or operational implications should be further validated in multicenter datasets with differing case-mix and care structures.

## Figures and Tables

**Table 1:** Annual CRP test volume and summary statistics, including arithmetic, harmonic, geometric means, median, and interquartile range (IQR), 2008–2023.

Year	Tests (*n*)	Arithmetic Mean (mg/dL)	Harmonic Mean (mg/dL)	Geometric Mean (mg/dL)	Median (mg/dL)	IQR (Q1–Q3)
2008	5015	3.79	0.14	0.79	0.95	0.14–4.63
2009	69,714	3.46	0.22	0.92	0.85	0.29–4.12
2010	80,931	3.25	0.18	0.83	0.81	0.30–3.92
2011	89,891	3.43	0.18	0.85	0.86	0.28–4.16
2012	98,896	3.24	0.19	0.84	0.84	0.29–4.14
2013	108,431	3.43	0.19	0.88	0.92	0.29–4.42
2014	114,091	3.27	0.18	0.80	0.78	0.22–4.10
2015	121,330	3.38	0.17	0.81	0.81	0.21–4.30
2016	137,726	3.41	0.18	0.82	0.84	0.21–4.16
2017	141,682	3.32	0.16	0.77	0.79	0.19–4.09
2018	147,864	3.46	0.17	0.80	0.79	0.19–4.24
2019	153,581	3.47	0.17	0.79	0.79	0.17–4.29
2020	143,811	3.41	0.16	0.76	0.79	0.16–4.21
2021	150,099	3.34	0.15	0.73	0.73	0.15–4.13
2022	169,697	3.38	0.17	0.76	0.72	0.15–4.20
2023	112,499	3.28	0.16	0.72	0.65	0.14–4.05

*n* indicates the total number of CRP test results recorded each year. Annual summary statistics are presented as the arithmetic mean, harmonic mean, and geometric mean of CRP concentrations (mg/dL).

**Table 2:** Weighted least squares regression of annual CRP summary measures (weights = annual test volume), 2008–2023.

Outcome (Annual Mean)	Slope, β (mg/dL per Year)	95% CI	R^2^	*p*-Value
Arithmetic mean (mg/dL)	+0.000765	−0.009955 to +0.011486	0.002	0.880
Harmonic mean (mg/dL)	−0.002716	−0.004050 to −0.001383	0.576	<0.001
Geometric mean (mg/dL)	−0.010504	−0.013654 to −0.007354	0.785	<0.001

Weighted least squares regression models were fitted to annual CRP summary measures, weighting each year by the annual number of CRP tests (weights = *N*) to reflect differences in the precision of annual estimates. β represents the estimated annual change (mg/dL per year) in each CRP summary measure. Calendar year was centered at 2008 (Year_c = Year − 2008) to improve interpretability of the intercept; this centering does not affect β or *p*-values. CI, confidence interval.

**Table 4:** Counterfactual deviations relative to the continuation of the pre-2020 trend.

Metric	Deviation in 2023 (mg/dL)	Cumulative Deviation, 2020–2023 (mg/dL·Year)
Arithmetic mean	−0.149	−0.349
Harmonic mean	+0.0118	+0.0139
Geometric mean	−0.0101	−0.0506

Weighted least squares (WLS) regression was fitted with annual test volume (*N*) as analytic weights. The ITS model included time (calendar year), a post-2020 indicator (level change), and time after 2020 (slope change). The post-2020 net slope was calculated as β_1_ + β_3_. Counterfactual deviations represent the difference between the fitted post-2020 trajectory and the counterfactual trajectory obtained by extending the pre-2020 trend through 2020–2023.

## Data Availability

The data underlying the results of this study originate from clinical records at Dankook University Hospital and are governed by ethical and legal regulations. To protect patient privacy and confidentiality, the original datasets are not publicly accessible. Nevertheless, de-identified aggregated data may be provided by the corresponding author upon reasonable request, contingent on approval from the Institutional Review Board.

## Supplementary Materials

The following supporting information can be downloaded at: https://www.mdpi.com/article/10.3390/diagnostics16071081/s1, Supplementary File S1: R scripts for statistical analyses; Table S1. Distribution of repeated CRP testing per patient in a 16-year hospital-based dataset (2008–2023). Table S2. Long-term temporal trends in annual CRP summary metrics after exclusion of individuals with more than 300 CRP measurements over the study period. Table S3. Sensitivity analysis excluding 2008: Weighted long-term linear trends in annual arithmetic, harmonic, and geometric mean CRP. Table S4. Quadratic sensitivity analysis for weighted annual trends in CRP summary metrics. Table S5. Multiplicity-adjusted inference for the post-2020 slope-change term (β_3_) across the three ITS summary metrics. Table S6. Sensitivity analyses of two-segment interrupted time-series (ITS) segmented regression of annual CRP summary measures using alternative interruption points at 2019 and 2021. Table S7. Sensitivity weighted interrupted time-series segmented regression of annual CRP summary measures after excluding individuals with more than 300 CRP measurements over the study period. Table S8. Counterfactual deviations from the continuation of the pre-2020 trend in the sensitivity interrupted time-series analysis after excluding individuals with more than 300 CRP measurements. Table S9. Sensitivity analysis excluding 2008: weighted interrupted time-series coefficients for annual arithmetic, harmonic, and geometric mean CRP. Table S10. Sensitivity analysis excluding 2008: deviation from the counterfactual trajectory in weighted interrupted time-series models of annual CRP summary metrics.

## Abbreviations

The following abbreviations are used in this manuscript:

CI	Confidence interval
CRP	C-reactive protein
ITS	Interrupted time series
WLS	Weighted least squares
HAC	Heteroskedasticity- and autocorrelation-consistent
QC	Quality control
IQR	Interquartile range
