# Supplementary material for: C-Reactive Protein Trajectories by Summary Metric Across the Coronavirus-2019 Period: A 16-Year Interrupted Time-Series Analysis (2008–2023)
Source: Diagnostics (Basel). 2026 Apr 3;16(7):1081. doi: 10.3390/diagnostics16071081 (PMC13073204; doi:10.3390/diagnostics16071081)
Supplement: Supplementary file 1 [file diagnostics-16-01081-s001.zip › CRP_annual_Supplementary_Tables.pdf]

Table S1 Distribution of repeated CRP testing per patient in a 16-year hospital-based dataset (2008–2023)

Number of CRP tests per patient (n)	Number of patients (n)
1	156,790
2	49,462
3	27,043
4	17,952
5	13,070
6	9753
7	7612
8	6020
9	4864
10	4191
11	3589
12	2944
13	2503
14	2206
15	2008
16	1793
17	1610
18	1406
19	1310
20	1178
21	1076
22	994
23	902
24	859
25	813
26	724
27	668
28	649
29	668
30	581
31	548
32	462
33	426
34	452
35	418

36	399
37	381
38	346
39	345
40	320
41	279
42	304
43	277
44	239
45	254
46	227
47	205
48	186
49	196
50	192
51	178
52	176
53	172
54	148
55	165
56	153
57	155
58	130
59	129
60	106
61	120
62	111
63	125
64	85
65	97
66	75
67	81
68	89
69	82
70	72
71	84
72	76
73	83
74	55

75	66
76	59
77	46
78	57
79	51
80	57
81	58
82	56
83	50
84	34
85	39
86	51
87	40
88	40
89	42
90	34
91	31
92	34
93	38
94	29
95	24
96	26
97	31
98	27
99	24
100	27
101	38
102	29
103	23
104	24
105	20
106	21
107	18
108	16
109	15
110	16
111	21
112	21
113	20

114	19
115	11
116	15
117	16
118	15
119	22
120	11
121	12
122	24
123	13
124	12
125	12
126	16
127	7
128	9
129	11
130	12
131	12
132	9
133	8
134	8
135	5
136	8
137	10
138	7
139	5
140	5
141	4
142	2
143	8
144	4
145	2
146	7
147	6
148	3
149	3
150	5
151	6
152	6

153	2
154	8
155	5
156	10
157	3
158	1
159	6
160	4
161	2
162	5
163	1
164	2
165	8
166	5
167	4
168	3
169	3
170	5
171	2
172	6
173	1
174	0
175	2
176	2
177	2
178	0
179	1
180	1
181	5
182	4
183	1
184	3
185	0
186	1
187	1
188	3
189	1
190	2
191	4

192	1
193	1
194	0
195	2
196	1
197	3
198	2
199	1
200	1
201	2
202	3
203	1
204	1
205	1
206	2
207	1
208	1
209	4
210	2
211	0
212	0
213	0
214	0
215	0
216	0
217	1
218	1
219	0
220	1
221	1
222	2
223	1
224	0
225	1
226	1
227	1
228	4
229	0
230	2

231	0
232	0
233	2
234	1
235	0
236	0
237	1
238	0
239	2
240	1
241	3
242	0
243	0
244	0
245	1
246	0
247	0
248	1
249	0
250	2
251	5
252	2
253	1
254	0
255	2
256	2
257	0
258	0
259	1
260	1
261	1
262	0
263	1
264	0
265	0
266	0
267	0
268	0
269	1

270	1
271	0
272	0
273	0
274	0
275	0
276	0
277	0
278	0
279	3
280	0
281	0
282	0
283	1
284	1
285	2
286	1
287	0
288	0
289	1
290	0
291	0
292	1
293	0
294	0
295	0
296	0
297	0
298	1
299	1
300	0
301	0
302	0
303	0
304	0
305	0
306	0
307	0
308	0

309	1
310	1
311	0
312	0
313	0
314	0
315	0
316	0
317	0
318	0
319	0
320	0
321	0
322	0
323	0
324	0
325	0
326	0
327	0
328	0
329	0
330	0
331	0
332	0
333	0
334	0
335	0
336	0
337	0
338	0
339	0
340	0
341	0
342	0
343	0
344	0
345	0
346	0
347	0

348	0
349	0
350	0
351	0
352	0
353	0
354	0
355	1
356	0
357	0
358	0
359	0
360	0
361	0
362	1
363	0
364	0
365	0
366	0
367	1
368	0
369	0
370	0
371	0
372	0
373	0
374	0
375	0
376	1
377	0
378	0
379	0
380	0
381	0
382	0
383	0
384	0
385	0
386	0

387	0
388	0
389	0
390	0
391	0
392	0
393	0
394	0
395	0
396	0
397	0
398	0
399	0
400	0
401	0
402	0
403	0
404	0
405	0
406	0
407	0
408	0
409	0
410	0
411	0
412	0
413	0
414	0
415	0
416	0
417	0
418	0
419	0
420	0
421	0
422	0
423	0
424	0
425	0

426	0
427	0
428	0
429	0
430	0
431	0
432	0
433	0
434	0
435	0
436	0
437	0
438	0
439	0
440	0
441	0
442	0
443	0
444	0
445	0
446	0
447	1
448	0
449	0
450	0
451	0
452	0
453	0
454	0
455	0
456	0
457	0
458	0
459	0
460	0
461	0
462	0
463	0
464	0

465	0
466	0
467	0
468	0
469	0
470	0
471	0
472	0
473	0
474	0
475	0
476	0
477	0
478	0
479	0
480	0
481	0
482	0
483	0
484	0
485	0
486	0
487	0
488	0
489	0
490	0
491	0
492	0
493	0
494	0
495	0
496	0
497	0
498	0
499	0
500	0
501	0
502	0
503	0

504	0
505	0
506	0
507	0
508	0
509	0
510	0
511	0
512	0
513	0
514	0
515	0
516	0
517	0
518	0
519	0
520	0
521	0
522	0
523	0
524	0
525	0
526	0
527	0
528	0
529	0
530	0
531	0
532	0
533	0
534	0
535	0
536	0
537	0
538	0
539	0
540	0
541	0
542	0

543	0
544	0
545	0
546	0
547	0
548	0
549	0
550	0
551	0
552	0
553	0
554	0
555	0
556	0
557	0
558	0
559	0
560	0
561	0
562	0
563	0
564	0
565	0
566	0
567	0
568	0
569	0
570	0
571	0
572	0
573	0
574	0
575	0
576	0
577	0
578	0
579	0
580	0
581	0

582	0
583	0
584	0
585	0
586	0
587	1
588	0
589	0
590	0
591	0
592	0
593	0
594	0
595	0
596	0
597	0
598	0
599	1
Total (n)	1,845,258

Abbreviations: CRP, C-reactive protein

Table S2. Long-term temporal trends in annual CRP summary metrics after exclusion of individuals with more than 300 CRP measurements over the study period.

Outcome	Slope, β (mg/dL per year)	95% CI	R^2	p-value
Arithmetic mean (mg/dL)	+0.000648	-0.010056 to +0.011352	0.001	0.899
Harmonic mean (mg/dL)	-0.002506	-0.003782 to -0.001231	0.559	<0.001
Geometric mean (mg/dL)	-0.010307	-0.013597 to -0.007018	0.763	<0.001

Table S3. Sensitivity analysis excluding 2008: Weighted long-term linear trends in annual arithmetic, harmonic, and geometric mean CRP.

Outcome	Slope, β (mg/dL per year)	95% CI	R^2	p-value
Arithmetic mean (mg/dL)	0.001385	-0.009403 to 0.012173	0.006	0.786
Harmonic mean (mg/dL)	-0.002799	-0.004132 to -0.001466	0.613	<0.001
Geometric mean (mg/dL)	-0.010649	-0.013872 to -0.007426	0.797	<0.001

Weighted least squares models were refitted after excluding the year 2008. β represents the annual change in each CRP summary metric, weighted by annual test volume.

Table S4. Quadratic sensitivity analysis for weighted annual trends in CRP summary metrics

**Outcome**	**Linear slope (β)**	**Linear p-value**	**Quadratic term (β2)**	**Quadratic p-value**	**Linear vs quadratic comparison p-value**	**Interpretation**
Arithmetic mean	0.0008	0.881	-0.00082	0.546	0.546	unchanged
Harmonic mean	-0.0027	<0.001	0.00017	0.299	0.299	unchanged
Geometric mean	-0.0105	<0.001	-0.00005	0.901	0.901	unchanged

Table S5. Multiplicity-adjusted inference for the post-2020 slope-change term (β3) across the three ITS summary metrics

Metric	Estimate (β3)	Raw p-value	Bonferroni-adjusted p-value	BH-adjusted p-value	Interpretation
Arithmetic mean	-0.0414	0.00549	0.0165	0.0165	Remained significant after correction
Harmonic mean	+0.00557	0.0409	0.1227	0.0614	No longer significant after correction
Geometric mean	+0.00167	0.760	1.0000	0.760	Not significant

Table S6. Sensitivity analyses of two-segment interrupted time-series (ITS) segmented regression of annual CRP summary measures using alternative interruption points at 2019 and 2021

Breakpoint	Metric	Pre-break slope β_1 (time)	p-value	Immediate level change β_2 (post-break indicator)	95% CI	p-value	Post-break slope change β_3 (time-after-break)	95% CI	p-value	Post-break net slope (β_1+β_3)	95% CI	p-value
2019	Arithmetic mean	+0.00478	0.521	+0.0647	−0.00470 to +0.134	0.093	−0.0444	−0.0645 to −0.0243	<0.001	−0.0396	−0.0514 to −0.0278	<0.001
	Harmonic mean	−0.00355	0.006	+0.00220	−0.00869 to +0.0131	0.699	+0.00264	−0.00165 to +0.00694	0.251	−0.000904	−0.00440 to +0.00259	0.613
	Geometric mean	−0.0103	<0.001	+0.00534	−0.0165 to +0.0271	0.640	−0.00271	−0.0120 to +0.00659	0.476	−0.0130	−0.0209 to −0.00520	0.001
2021	Arithmetic mean	−0.00310	0.731	−0.0678	−0.146 to +0.0104	0.119	−0.0340	−0.0800 to +0.0120	0.170	−0.0371	−0.0852 to +0.0109	0.130
	Harmonic mean	−0.00116	0.437	−0.00307	−0.0147 to +0.00859	0.652	+0.00896	−0.00100 to +0.0189	0.092	+0.00780	−0.00107 to +0.0167	0.085
	Geometric mean	−0.00819	0.002	−0.0158	−0.0422 to +0.0108	0.275	+0.00704	−0.0157 to +0.0298	0.562	−0.00115	−0.0227 to +0.0204	0.916

Annual arithmetic, harmonic, and geometric mean CRP concentrations (mg/dL) were modeled using a two-segment ITS framework with alternative interruption points at 2019 and 2021. The model included a continuous time term (β_1; pre-break slope), a post-break indicator (β_2; immediate level change at the interruption year), and a time-after-break term (β_3; change in slope after the interruption year). The post-break net slope was calculated as β_1+β_3 and is reported with its 95% confidence interval. Weighted least squares (WLS) regression was fitted using annual test volume (N) as analytic weights.

Table S7. Sensitivity weighted interrupted time-series segmented regression of annual CRP summary measures after excluding individuals with more than 300 CRP measurements over the study period

Metric	Parameter	Estimate (β)	95% CI	p-value
Arithmetic mean	β1: Pre-2020 slope	+0.00923	-0.00774 to +0.0262	0.259
	β2: Level change at 2020	-0.0315	-0.198 to +0.135	0.688
	β3: Slope change after 2020	-0.0399	-0.115 to +0.0349	0.268
	Post-2020 net slope (β1 + β3)	-0.0307	-0.103535 to +0.042173	0.377
Harmonic mean	β1: Pre-2020 slope	-0.00262	-0.00465 to -0.000580	0.016
	β2: Level change at 2020	-0.00932	-0.0293 to +0.0107	0.330
	β3: Slope change after 2020	+0.00629	-0.00269 to +0.0153	0.153
	Post-2020 net slope (β1 + β3)	+0.00367	-0.005069 to +0.012413	0.378
Geometric mean	β1: Pre-2020 slope	-0.00890	-0.0145 to -0.00329	0.005
	β2: Level change at 2020	-0.0194	-0.0745 to +0.0357	0.458
	β3: Slope change after 2020	+0.00192	-0.0228 to +0.0266	0.869
	Post-2020 net slope (β1 + β3)	-0.00698	-0.031054 to +0.017085	0.539

Annual arithmetic, harmonic, and geometric mean CRP concentrations (mg/dL) were modeled using a two-segment interrupted time-series framework with 2020 as the prespecified interruption. The model included a continuous time term (β_1; pre-2020 slope), a post-2020 indicator (β_2; immediate level change at 2020), and a time-after-2020 term (β_3; change in slope after 2020). The post-2020 net slope was calculated as $\beta_1+\beta_3$. Weighted least squares regression was fitted using annual test volume as analytic weights after excluding individuals with more than 300 CRP measurements over the study period.

Table S8. Counterfactual deviations from the continuation of the pre-2020 trend in the sensitivity interrupted time-series analysis after excluding individuals with more than 300 CRP measurements

Metric	Deviation in 2023 (mg/dL)	Cumulative deviation, 2020–2023 (mg/dL·year)
Arithmetic mean	-0.151	-0.365
Harmonic mean	+0.00955	+0.000462
Geometric mean	-0.0136	-0.0660

Counterfactual deviations represent the difference between the fitted post-2020 trajectory from the weighted interrupted time-series model and the counterfactual trajectory obtained by extending the pre-2020 trend through 2020–2023. The deviation in 2023 corresponds to the model-based difference in 2023, and the cumulative deviation represents the sum of annual deviations across 2020–2023. Analyses were performed after excluding individuals with more than 300 CRP measurements over the study period.

Table S9. Sensitivity analysis excluding 2008: weighted interrupted time-series coefficients for annual arithmetic, harmonic, and geometric mean CRP

Metric	Parameter	Estimate (β)	SE	95% CI	p-value
Arithmetic mean	β1: Pre-2020 slope	0.010349	0.006395	-0.002185 to 0.022883	0.134
	β2: Level change at 2020	-0.030338	0.041187	-0.111063 to 0.050388	0.477
	β3: Slope change after 2020	-0.042721	0.012024	-0.066287 to -0.019155	0.005
	Post-2020 net slope (β1 + β3)	-0.032372	0.009854	-0.051686 to -0.013058	0.001
Harmonic mean	β1: Pre-2020 slope	-0.003275	0.001082	-0.005394 to -0.001155	0.011
	β2: Level change at 2020	-0.004225	0.005685	-0.015368 to 0.006918	0.473
	β3: Slope change after 2020	0.005738	0.002507	0.000823 to 0.010652	0.043
	Post-2020 net slope (β1 + β3)	0.002463	0.002164	-0.001778 to 0.006705	0.255
Geometric mean	β1: Pre-2020 slope	-0.009698	0.002150	-0.013912 to -0.005484	<0.001
	β2: Level change at 2020	-0.014099	0.012666	-0.038924 to 0.010726	0.289
	β3: Slope change after 2020	0.001934	0.005456	-0.008759 to 0.012628	0.730
	Post-2020 net slope (β1 + β3)	-0.007764	0.004899	-0.017366 to 0.001839	0.113

Table S10. Sensitivity analysis excluding 2008: deviation from the counterfactual trajectory in weighted interrupted time-series models of annual CRP summary metrics

Metric	Deviation in 2023 (mg/dL)	Cumulative deviation, 2020–2023 (mg/dL·year)
Arithmetic mean	-0.1590	-0.3780
Harmonic mean	0.0130	0.0175
Geometric mean	-0.0083	-0.0448
