# Supplementary material for: C-Reactive Protein Trajectories by Summary Metric Across the Coronavirus-2019 Period: A 16-Year Interrupted Time-Series Analysis (2008–2023)
Source: Diagnostics (Basel). 2026 Apr 3;16(7):1081. doi: 10.3390/diagnostics16071081 (PMC13073204; doi:10.3390/diagnostics16071081)
Supplement: Supplementary file 1 [file diagnostics-16-01081-s001.zip › Supplementary File S1. R_scripts.pdf]

Supplementary File S1. R scripts for statistical analyses

This file contains the R scripts used to reproduce the weighted least squares (WLS) regression analyses (Section 3.2) and the interrupted time-series (ITS) segmented regression analyses (Section 3.3) reported in the manuscript.

Script S1: Long-term WLS trend analysis (Section 3.2)

```
## ---- 0) packages ----
library(dplyr)
library(broom)
library(ggplot2)

## ---- 1) data ----
df <- tribble(
  ~Year, ~Tests,   ~Arithmetic_mean, ~Harmonic_mean, ~Geometric_mean,
  2008,    5015,    3.79,              0.14,           0.79,
  2009,   69714,    3.46,              0.22,           0.92,
  2010,   80931,    3.25,              0.18,           0.83,
  2011,   89891,    3.43,              0.18,           0.85,
  2012,   98896,    3.24,              0.19,           0.84,
  2013, 108431,    3.43,              0.19,           0.88,
  2014, 114091,    3.27,              0.18,           0.80,
  2015, 121330,    3.38,              0.17,           0.81,
  2016, 137726,    3.41,              0.18,           0.82,
  2017, 141682,    3.32,              0.16,           0.77,
  2018, 147864,    3.46,              0.17,           0.80,
  2019, 153581,    3.47,              0.17,           0.79,
  2020, 143811,    3.41,              0.16,           0.76,
  2021, 150099,    3.34,              0.15,           0.73,
  2022, 169697,    3.38,              0.17,           0.76,
  2023, 112499,    3.28,              0.16,           0.72
) %>%
  mutate(Year_c = Year - 2008)   # centering (intercept = 2008)

## ---- 2) WLS models (weights = Tests) ----
fit_arith <- lm(Arithmetic_mean ~ Year_c, data = df, weights = Tests)
fit_harm   <- lm(Harmonic_mean     ~ Year_c, data = df, weights = Tests)
fit_geom   <- lm(Geometric_mean   ~ Year_c, data = df, weights = Tests)

## ---- 3) Table 2 style summary ----
```

```
extract_row <- function(fit, outcome_label){
  s <- summary(fit)
  ci <- confint(fit)["Year_c", ]
  tibble(
    Outcome = outcome_label,
    beta = unname(coef(fit)["Year_c"]),
    CI_low = unname(ci[1]),
    CI_high = unname(ci[2]),
    R2 = s$r.squared,
    p_value = coef(s)["Year_c", "Pr(>|t|)"]
  )
}

tab2 <- bind_rows(
  extract_row(fit_arith, "Arithmetic mean (mg/dL)"),
  extract_row(fit_harm,    "Harmonic mean (mg/dL)"),
  extract_row(fit_geom,    "Geometric mean (mg/dL)")
) %>%
  mutate(
    beta = sprintf("%.6f", beta),
    `95% CI` = sprintf("%+.6f to %+.6f", CI_low, CI_high),
    R2 = sprintf("%.3f", R2),
    p_value = ifelse(p_value < 0.001, "<0.001", sprintf("%.3f", p_value))
  ) %>%
  select(Outcome, `Slope, β (mg/dL per year)` = beta, `95% CI`, R2, `p-value` = p_value)

print(tab2)
```

Script S2: Interrupted time-series analysis (Section 3.3)

```
## ---------------------------
## 0) packages
## ---------------------------
library(dplyr)
library(tidyr)
library(broom)
library(lmtest)
library(sandwich)
library(ggplot2)
```

```r
## ---------------------------
## 1) data
## ---------------------------
df <- tibble::tribble(
  ~Year, ~Tests,   ~Arithmetic_mean, ~Harmonic_mean, ~Geometric_mean,
  2008,    5015,        3.79,              0.14,             0.79,
  2009,   69714,        3.46,              0.22,             0.92,
  2010,   80931,        3.25,              0.18,             0.83,
  2011,   89891,        3.43,              0.18,             0.85,
  2012,   98896,        3.24,              0.19,             0.84,
  2013, 108431,        3.43,              0.19,             0.88,
  2014, 114091,        3.27,              0.18,             0.80,
  2015, 121330,        3.38,              0.17,             0.81,
  2016, 137726,        3.41,              0.18,             0.82,
  2017, 141682,        3.32,              0.16,             0.77,
  2018, 147864,        3.46,              0.17,             0.80,
  2019, 153581,        3.47,              0.17,             0.79,
  2020, 143811,        3.41,              0.16,             0.76,
  2021, 150099,        3.34,              0.15,             0.73,
  2022, 169697,        3.38,              0.17,             0.76,
  2023, 112499,        3.28,              0.16,             0.72
)

## ---------------------------
## 2) ITS design variables (2020 interruption)
## ---------------------------
df <- df %>%
  arrange(Year) %>%
  mutate(
    time = Year - min(Year),                          # 2008=0, 2009=1, ...
    post2020 = ifelse(Year >= 2020, 1, 0),        # level change indicator
    time_after_2020 = pmax(0, Year - 2020)        # slope change term: 2020=0, 2021=1...
  )
fit_its_wls <- function(data, yvar, use_hac = TRUE, hac_lag = 1) {
  fml <- as.formula(paste0(yvar, " ~ time + post2020 + time_after_2020"))
  mod <- lm(fml, data = data, weights = Tests)    # WLS with weight=Tests

  # robust SE (HAC) or conventional
```

```
V <- if (use_hac) {
  sandwich::NeweyWest(mod, lag = hac_lag, prewhite = FALSE, adjust = TRUE)
} else {
  vcov(mod)
}

ct <- lmtest::coeftest(mod, vcov. = V)

# tidy coefficient table
coef_tab <- tibble(
  term = rownames(ct),
  estimate = ct[, 1],
  std.error = ct[, 2],
  statistic = ct[, 3],
  p.value = ct[, 4]
) %>%
  mutate(
    conf.low   = estimate - 1.96 * std.error,
    conf.high = estimate + 1.96 * std.error
  )

# post net slope = beta_time + beta_time_after_2020
b <- coef(mod)
# linear combination
L <- c(0, 1, 0, 1) # (Intercept, time, post2020, time_after_2020)
post_slope <- sum(L * b)

post_slope_se <- as.numeric(sqrt(t(L) %*% V %*% L))
post_slope_ci <- c(post_slope - 1.96 * post_slope_se, post_slope + 1.96 * post_slope_se)
post_slope_p   <- 2 * (1 - pnorm(abs(post_slope / post_slope_se)))

post_slope_tab <- tibble(
  term = "post_net_slope (beta_time + beta_time_after_2020)",
  estimate = post_slope,
  std.error = post_slope_se,
  conf.low = post_slope_ci[1],
  conf.high = post_slope_ci[2],
  p.value = post_slope_p
)
```

```r
  # counterfactual predictions: set post2020=0 and time_after_2020=0 for all years
  data_cf <- data %>% mutate(post2020 = 0, time_after_2020 = 0)

  pred_obs <- predict(mod, newdata = data,     se.fit = FALSE)
  pred_cf   <- predict(mod, newdata = data_cf, se.fit = FALSE)

  out_df <- data %>%
    transmute(
      Year, Tests,
      y = .data[[yvar]],
      fitted = pred_obs,
      counterfactual = pred_cf,
      deviation = fitted - counterfactual
    )

  dev_2023 <- out_df %>% filter(Year == 2023) %>% pull(deviation)
  dev_cum   <- out_df %>% filter(Year >= 2020) %>% summarise(cum = sum(deviation)) %>% pull(cum)

  list(
    model = mod,
    coef_table = bind_rows(coef_tab, post_slope_tab),
    fitted_table = out_df,
    dev_2023 = dev_2023,
    dev_cum_2020_2023 = dev_cum
  )
}
metrics <- c("Arithmetic_mean", "Harmonic_mean", "Geometric_mean")

fits <- lapply(metrics, function(m) fit_its_wls(df, m, use_hac = TRUE, hac_lag = 1))
names(fits) <- metrics

## (A) Table: model coefficients (β1, β2, β3, post net slope) summary
table_main <- bind_rows(lapply(names(fits), function(m) {
  fits[[m]]$coef_table %>%
    filter(term %in% c("time", "post2020", "time_after_2020",
                       "post_net_slope (beta_time + beta_time_after_2020)")) %>%
    mutate(metric = m) %>%
    select(metric, term, estimate, std.error, conf.low, conf.high, p.value)
```

```r
}))

table_main

## (B) Summary of deviations (2023 / cumulative 2020–2023)
dev_summary <- tibble(
  metric = names(fits),
  deviation_2023 = sapply(fits, \(x) x$dev_2023),
  cumulative_deviation_2020_2023 = sapply(fits, \(x) x$dev_cum_2020_2023)
)

dev_summary
```
